# Immune-mediated myositis following gene therapy for Duchenne muscular dystrophy: a case report

**DOI:** 10.1007/s00415-024-12431-z

**Published:** 2024-06-22

**Authors:** Susan T. Iannaccone, Chunyu Cai, Brittney Rhem, Kaitlin Batley, Veena Rajaram, Benjamin M. Greenberg, Sachi Dharia, Craig M. Zaidman

**Affiliations:** 1 Departments of Pediatrics and Neurology, University of Texas Southwestern Medical Center and Children’s Health, Dallas, TX USA; 2 Department of Pathology, University of Texas Southwestern Medical Center and Children’s Health, Dallas, TX USA; 3 Department of Neurology, University of Texas Southwestern Medical Center and Children’s Health, Dallas, TX USA; 4 Sarepta Therapeutics, Inc., Cambridge, MA USA; 5 Department of Neurology, Washington University, St. Louis, MO USA

**Dear Sirs,**

Duchenne muscular dystrophy (DMD) is caused by pathogenic variants in the *DMD* gene leading to an absence of functional dystrophin [1, 2]. Delandistrogene moxeparvovec, a recombinant adeno-associated viral vector (rAAV)-based gene therapy (GT) designed to compensate for the absence of dystrophin in DMD, delivers a transgene encoding delandistrogene moxeparvovec micro-dystrophin, an engineered dystrophin that retains key functional domains of the wild-type protein. In June 2023, delandistrogene moxeparvovec received accelerated approval for the treatment of ambulatory pediatric patients aged 4 through 5 years with a confirmed *DMD* gene mutation in the United States [3]. As of April 2024, delandistrogene moxeparvovec is approved in the United Arab Emirates, Qatar, Kuwait, Bahrain, and Oman for the same indication. Challenges to GT include the large size of the *DMD* gene which limits packaging ability into AAV vectors, the need for distribution to skeletal and cardiac muscles, localization to the sarcolemma, and overcoming immune responses to viral vectors or transgene protein products [4, 5].

The first experience treating DMD with an AAV serotype 2.5 (AAV2.5) vector-based mini-dystrophin transgene was reported in six patients. Three patients demonstrated T-cell response to dystrophin while none showed serum dystrophin-specific antibodies. Two patients appeared to have T-cells targeting dystrophin prior to treatment, suggestive of prior “priming” by revertant dystrophin fibers [6].

To date, six cases of immune-mediated myositis (IMM) have been disclosed post-GT with different AAV vector-based micro- or mini-dystrophin transgenes in clinical trials: five in patients with large overlapping deletions in the *DMD* gene [7] and one in a patient with a deletion of exons 8 and 9 [7, 8]. Mapping the epitopes encoded by exons 8–11 revealed the absence of hinge 1 in all five patients, suggesting it was a possible immunogenic target [7]. To mitigate events of IMM, delandistrogene moxeparvovec is contraindicated for patients with any deletion in exon 8 and/or exon 9 in the *DMD* gene [3]. This letter to the editor presents further details of the clinical course and biopsy for one of the patients who developed IMM after treatment with delandistrogene moxeparvovec.

## Methods

Clinical data were from the authors' observations and the electronic medical records of Children’s Health. Pre-treatment histologic samples were provided by Sarepta Therapeutics. The muscle biopsy was processed at the University of Texas Southwestern Medical Center.

### Case presentation

**Fig. 1:**
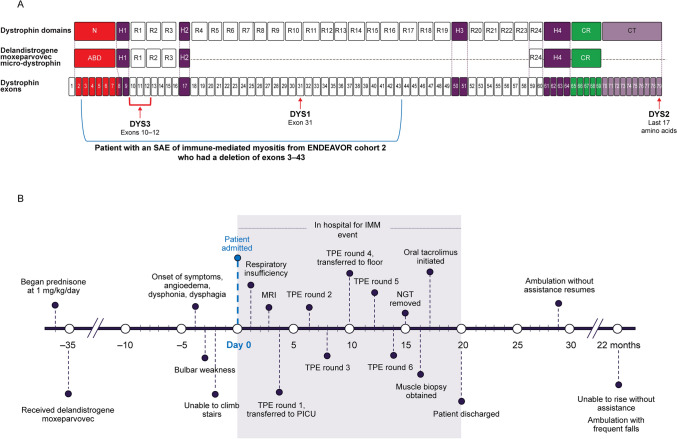
Deletion of exons 3–43 of the patient’s *DMD* gene and timeline of the IMM event. **A** Deletion of exons 3–43 of the patient’s *DMD* gene. **B** Timeline of the IMM event. Abbreviations: ABD = actin-binding domain; CR = cysteine-rich domain; CT = C-terminal domain; DMD, Duchenne muscular dystrophy; DYS = dystrophin; H = hinge domain; IMM = immune-mediated myositis; MRI = magnetic resonance imaging; N = N-terminal; NGT = nasogastric tube; PICU = pediatric intensive care unit; R = spectrin‑like repeat domain; SAE = serious adverse event; TPE = therapeutic plasma exchange

A 9-year-old ambulatory male patient, diagnosed with DMD at 2.5 years of age presented with acute-onset bulbar and limb girdle weakness 1 month post-treatment with delandistrogene moxeparvovec GT in ENDEAVOR (SRP-9001-103, NCT04626674) cohort 2 (Fig. 1A). This patient has a deletion of exons 3–43 in the *DMD* gene, which encodes part of the N-terminal, part of the central rod domain, and hinge 1 and hinge 2. Parents described onset of symptoms 4 days earlier with swelling of the lips and lower face, and change in voice; 2 days later, he was unable to climb stairs (Fig. 1B). Pre-GT, the patient was maintained on daily deflazacort (27 mg/day [0.9 mg/kg/day]) with good motor function, independent ambulation, and no history of respiratory insufficiency or heart disease. Daily prednisone (30 mg/day [1 mg/kg/day]) was added to his regimen 24-h pre-GT and was continued without any missed doses at the time of presentation. Other daily medications included vitamin D (1,000 IU), lisinopril (5 mg), fish oil (1200 mg), two multivitamins, coenzyme Q10 (100 mg), and melatonin (3 mg). He took alendronate (35 mg) weekly.

The patient was lethargic with angioedema, had increased work of breathing, hypophonic voice, and difficulty swallowing. He required help sitting and could only bear weight with assistance. Weakness was most severe in shoulder abductors and hip flexors. Deep tendon reflexes were intact at the Achilles bilaterally and absent at biceps, triceps, brachioradialis, and patella. He had no observed sensory deficit, muscle tenderness, or skin rash. Initial laboratory data showed no abnormality in complete blood count, erythrocyte sedimentation rate, venous capillary gases, electrolytes or troponin-I level (24 pg/ml), or creatine kinase (27,903 U/L); additional data collected at admission are shown in Online Resource Table 1. Due to worsening bulbar weakness, he was transferred to the intensive care unit and a nasogastric tube was placed for feeding. Heated high flow nasal cannula, then bilevel positive airway pressure, was administered for ventilation. Six rounds of therapeutic plasma exchange (TPE) were initiated [9]. After round four, he showed objective improvement in voice quality, could count to 10 without taking a breath, and could sit independently; thereafter, he showed slow but steady improvement. Following TPE, muscle biopsy was obtained from the left quadriceps (51 days post-GT; Fig. 1B). Three days before discharge (52 days post-GT), maintenance immunosuppression with oral tacrolimus was initiated and maintained for 27 months before tapering to cessation over 6 weeks. At discharge (20 days post-admission and 55 days post-GT), his creatine kinase level was 1,218 U/L and he could ambulate with slight assistance, tolerate all intake by mouth, and required no respiratory support.

Motor function assessments were conducted bi-annually during standard of care visits (Online Resource Table 2). During the last clinic visit (22 months post-GT), he was unable to rise independently and could ambulate for short distances with frequent falls. Pulmonary evaluation indicated normal respiratory muscle strength. Manual motor testing revealed lower proximal muscle strength compared with 1 year prior (10 months post-GT). Muscle biopsy showed minimal micro-dystrophin expression (12 months post-GT; Online Resource Table 3).

## Results

### Muscle histology

**Fig. 2:**
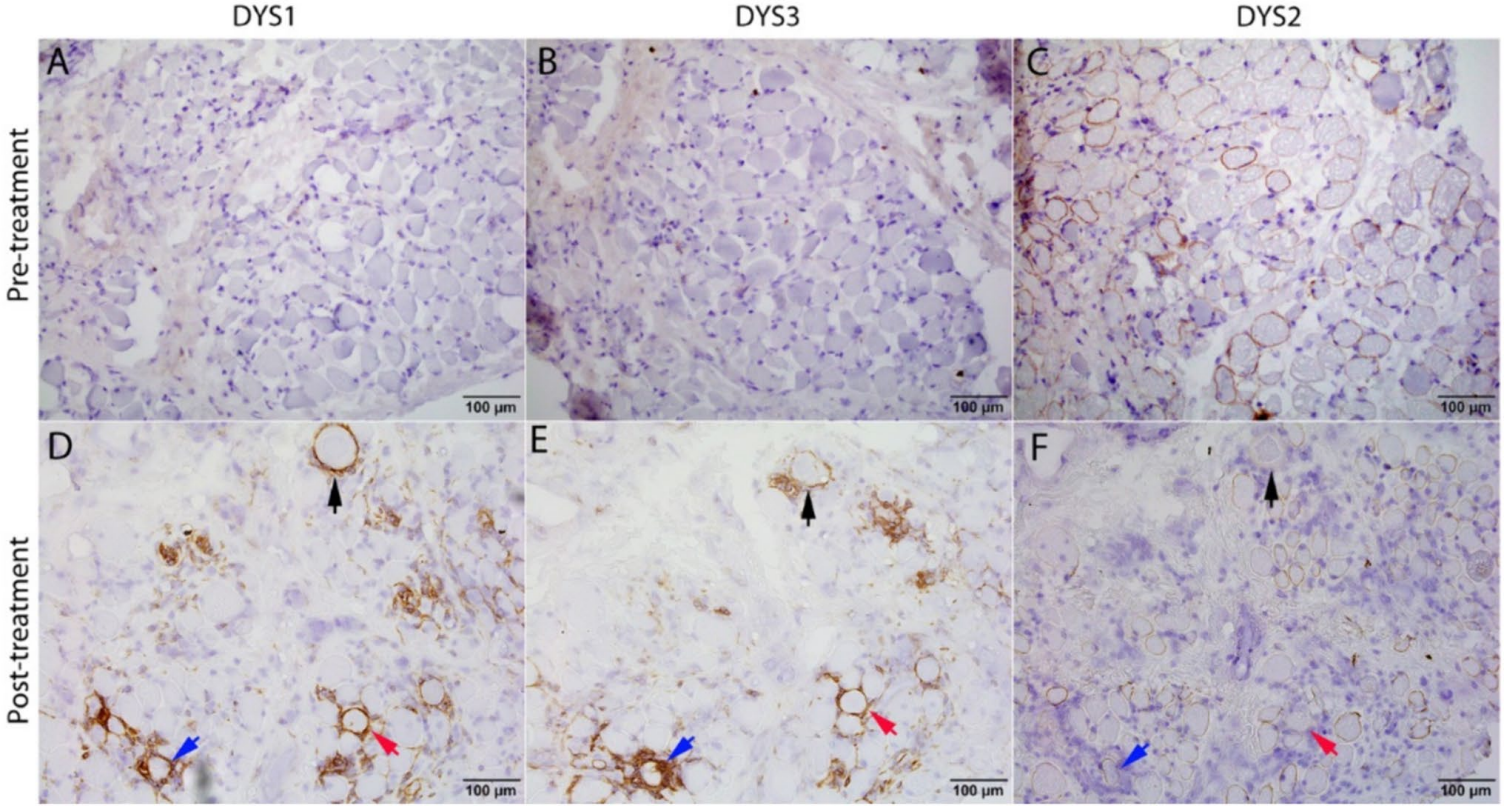
Dystrophin epitope staining in pre- and post-treatment muscle biopsies. Pre-treatment muscle demonstrated absence of **A** DYS1, **B** DYS3, and **C** Partial expression of DYS2 in some myofibers. In the post-treatment muscle biopsy, **D** DYS1 and **E** DYS3 were expressed in a subset of myofibers, the majority of which were under attack by inflammatory cells. **F** DYS2 was expressed in a subset of myofibers similar to pre-treatment muscle. Images of **D**–**F** were taken from the same field on serial sections. The color-coded arrows (black, blue, and red) point out three myofibers on serial sections with different epitope stains. Scale bar: 100 µm. Abbreviation: DYS = dystrophin

**Fig. 3:**
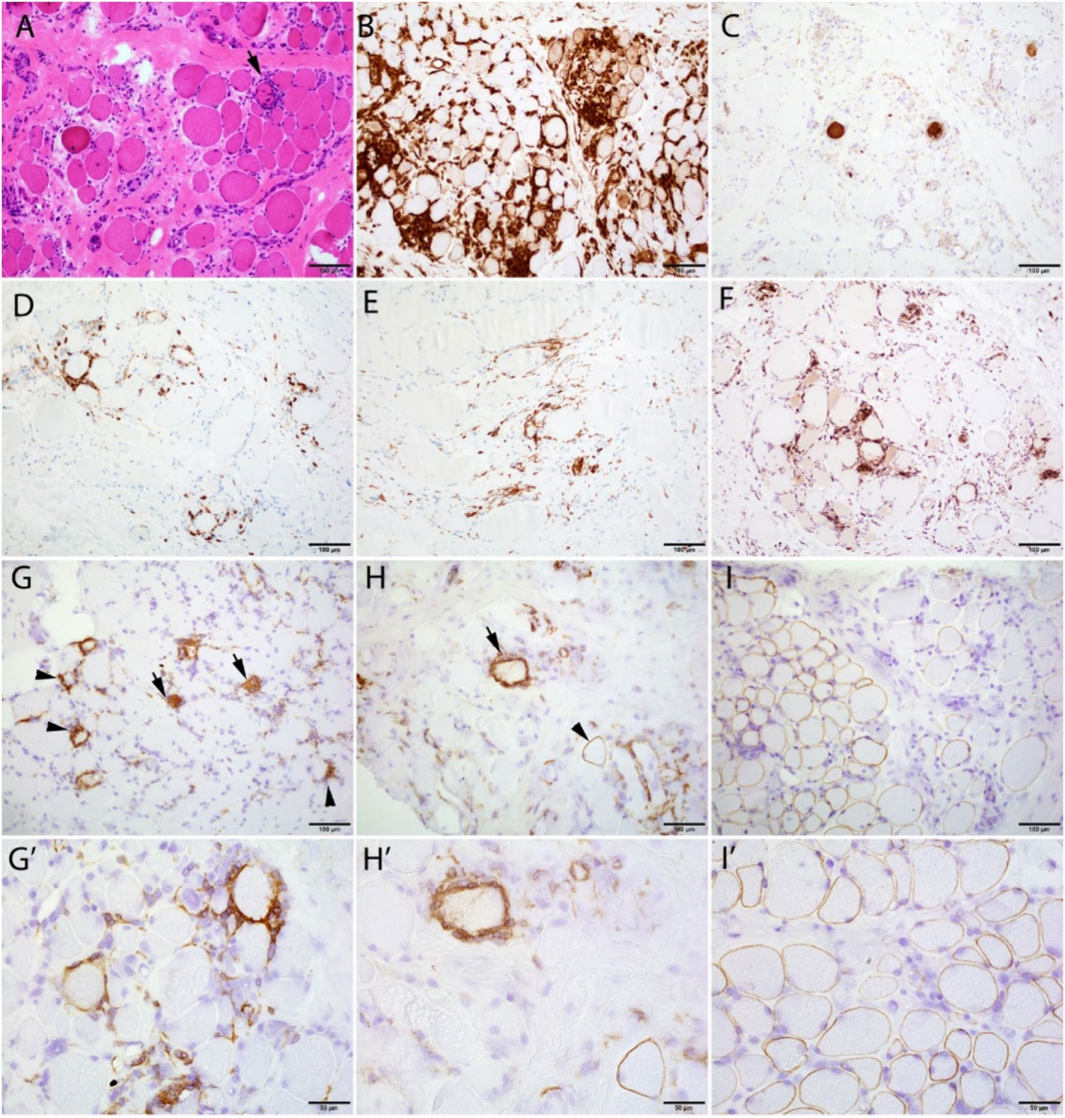
Post-treatment muscle histology. **A–I** Low magnification; scale bar: 100 µm. **A** H&E showed a muscle with chronic active dystrophic changes and endomysial mononuclear inflammation surrounding and invading viable myofibers (arrow). **B** MHC class I immunostain highlighted inflammatory cells and patchy MHC1 upregulation in myofibers. **C**. C5b-9 immunostain highlighted scattered necrotic fibers. **D–F** CD markers. **D** CD8. **E** CD4. **F** CD68. The mononuclear inflammation was composed of mixed CD8+ T cells, CD4+ T cells, and CD68+ macrophages. CD20+ B cells were absent (data not shown). **G**–**I** Immunostain of dystrophin epitopes. **G′–I′** High magnification; scale bar: 50 µm. **G**, **G**′ DYS1 (rod domain) was only expressed in a small subset of fibers, the vast majority of which were either dying (arrows) or under attack by inflammatory cells (arrow heads and **G′**). **H**, **H**′ DYS3 (N-terminal) was expressed in a small subset of myofibers, most of which were under attack by inflammatory cells (arrow); rare ones had intact sarcolemmal expression (arrowhead). (I, I’) DYS2 (C-terminal) was variably expressed in a significant subset of myofibers, all of which had an intact sarcolemmal membrane. Abbreviations: DYS = dystrophin; H&E = hematoxylin and eosin; MHC, major histocompatibility complex

In the biopsy obtained 1 day pre-treatment, dystrophin epitope immunohistochemical staining showed that a substantial subset (30–40%) of muscle fibers expressed a weak sarcolemmal C-terminal (dystrophin 2 [DYS2]; Fig. 2C) while rod domain (dystrophin 1 [DYS1]) and N-terminal (dystrophin 3 [DYS3]; Fig. 2B) were completely absent, suggesting that a truncated dystrophin protein containing the C-terminal was present pre-GT. In the post-treatment biopsy obtained after TPE, there was an abundance of CD8- and CD4-positive T cells and CD68-positive macrophages in the endomysium that surrounded and invaded individual myofibers (Fig. 3). These inflammatory cells appeared to selectively attack myofibers expressing the rod domain (Fig. 2D [arrows]) and transgene-derived N-terminal (Fig. 2E [arrows]), but not those expressing the native C-terminal (Fig. 2F).

## Discussion

Delandistrogene moxeparvovec transgene encodes for key functional domains of dystrophin including the N-terminal region [10], but not the most distal sequence (C-terminal) which includes the DYS2 epitope. Thus, in this patient’s post-treatment muscle, any C-terminal expression was native while any N-terminal expression was likely transgenic. This may explain why inflammatory cells were seemingly attacking myofibers reactive for DYS3, but not fibers reactive for DYS2 (Fig. 2). The sarcolemmal expression of the rod domain in the post-treatment muscle was unexpected as the sequence encoding the DYS1 epitope (exon 31 encoding R10 domain) was not included in the transgene construct (Fig. 1). We suspect that the DYS1 immunohistochemistry might not be specific to the R10 domain and bound to the transgene-derived protein as a majority of myofibers positive for DYS1 were positive for DYS3 (Fig. 2 [arrows]). Our findings may be indicative of T cell-mediated rejection against the delandistrogene moxeparvovec micro-dystrophin protein [11]. There was no B-cell inflammation (data not shown), arguing against antibody-mediated rejection [12]. Muscle biopsy was obtained after TPE; if present, the levels of pathogenic antibody would be decreased. Additionally, immunosuppression with steroids may have impacted the pathologic findings. Muscle biopsy was taken after corticosteroids had been administered, which could have altered the profile of lymphocytes present.

Given that AAV vectors serve as adjuvants, AAV-based GT carries the risk for humoral and cellular immune responses against novel proteins. This case shows an increased risk for severe IMM requiring hospitalization after treatment with delandistrogene moxeparvovec, in patients with deletion in exon 8 and/or exon 9 in the *DMD* gene. Further, our patient showed no treatment benefit, with ambulation not returning to baseline ability; however, this ongoing slow decline in ambulation is typical of DMD natural history. At 11 years old, he remains ambulatory, utilizing a wheelchair for long distances.

## Limitations of the study

This is a single case report; the results and conclusions presented herein may not be generalizable for patients who develop IMM following GT. The risk for developing IMM in patients with any deletion in exon 8 and/or exon 9 in the *DMD* gene warrants further investigation, specifically around contributing risk factors. Ongoing research is underway to better understand risk factors and to safely administer delandistrogene moxeparvovec in patients with potentially higher-risk *DMD* gene mutations.

### Supplementary Information

Below is the link to the electronic supplementary material.

Supplementary file1 (DOCX 30 kb)

## Data Availability

Anonymized data not published within this article will be made available by request to the corresponding author.
