# Supplementary material for: Immune-mediated myositis following gene therapy for Duchenne muscular dystrophy: a case report
Source: J Neurol. 2024 Jun 22;271(8):5659–64. doi: 10.1007/s00415-024-12431-z (PMC11319415; doi:10.1007/s00415-024-12431-z)
Supplement: Supplementary file 1 — Supplementary file1 (DOCX 30 kb) [file 415_2024_12431_MOESM1_ESM.docx]

**Online Resource**

**Methods**
Muscle was processed in a Clinical Laboratory Improvement Amendments approved clinical laboratory. Fresh muscle specimen was oriented, snap-frozen in isopentane-cooled liquid nitrogen and submitted for frozen section histology. Immunohistochemical staining for major histocompatibility complex class I (MHC1, US Biological, Salem, MA, M3886-10), C5b-9 (Dako, Santa Clara, CA, M0777) and dystrophin epitopes (DYS2, DYS3, Leica Biosystems, Buffalo Grove, IL) were performed on frozen sections. Additional segments of skeletal muscle were received isometrically fixed in 10% formalin and processed for paraffin embedding, hematoxylin and eosin histology. Immunohistochemical staining for CD4 (Ventana, Tuson, AZ, SP35), CD8 (Ventana, Tuson, AZ SP57), CD20 (Ventana, Tuson, AZ, L26), and CD68 (Ventana, Tuson, AZ, KP-1) were performed on paraffin sections. Immunohistochemistry was performed on a Ventana Benchmark Ultra automated immunostainer, using a Ventana UltraView Universal DAB Detection Kit. Delandistrogene moxeparvovec micro-dystrophin expression was assessed by droplet digital polymerase chain reaction (ddPCR) (vector genome copies) and western blot (WB) on patient biopsies obtained at baseline, week 7 and week 52; immunofluorescence percent dystrophin-positive fibers (IF PDPF) were measured at baseline and week 52. The ddPCR, WB and IF PDPF assays were conducted according to the methods described by Zaidman et al [1].

**Results**

**Table 1.** Initial laboratory data attributes upon admission of patient.

| **Parameter measured** | **Levels (range)** |
| --- | --- |
| Troponin-I | 24 pg/ml |
| CK | 27,903 U/L (21–232) |
| ALT | 1,400 U/L (10–50) |
| AST | 1,120 U/L (10–45) |
| GGT | 24 U/L (7–30) |
| Routine urinalysis | Normal |
| Urine toxicology screening | Negative |
| CMV IgG and IgM | Negative |
| EBV PCR | Negative |
| IgG and IgM | Negative |
| Cerebrospinal fluid  White blood cells  Protein | Clear  29 (17% monocytes, 83% lymphocytes)  16.6 mg/dL (10–45) |
| MRI of brain and spine | Widespread and multifocal myoedema but no intracranial, intrathecal, or spinal cord abnormalities |

Abbreviations: ALT = alanine aminotransferase; AST = aspartate transaminase; CK = creatine kinase, CMV = cytomegalovirus; EBV = Epstein-Barr virus; GGT = gamma-glutamyl transferase; IgG = immunoglobulin G; IgM = immunoglobulin M; MRI = magnetic resonance imaging; PCR = polymerase chain reaction

**Table 2.** Motor functions scores and observations of clinicians during the patient's standard of care visits.

| **Standard of care visit*** | **NSAA** | **10-Meter Walk/Run, seconds** | **PUL 2.0** | **Clinician notes** |
| --- | --- | --- | --- | --- |
| 2 months post-GT | – | – | 38 | NSAA was not performed; using a rolling walker |
| 10 months post-GT | 14 | 7.5 | – | Uses external support to rise from floor |
| 22 months post-GT | 10 | 8.5 | – | Cannot walk longer distances; uses external support to rise from floor |
| 29 months post-GT | 8 | 9.6 | 28 | Unable to climb/descend a box step |

The patient was 9 years old at baseline. NSAA, 10-Meter Walk/Run, and PUL 2.0 at baseline were measured within the clinical trial and data are not available. Abbreviations: GT = gene therapy; NSAA = North Star Ambulatory Assessment; PUL = Performance Upper Limb

**Table 3.** Patient biopsy data at baseline, week 7, and week 52.

| **Metric** | **Baseline** | **Week 7** | **Week 52** |
| --- | --- | --- | --- |
| Vector genome copies (ddPCR) | 0 | 1.77 | 0.46 |
| Western blot | Below LLOQ  No 137 kDa band | Below LLOQ  +137 kDa band* | Below LLOQ  +137 kDa band |
| IF PDPF | 0.38% |  | 2.33% |

*No 137 kDa band in western blot. When tested in ProteinSimple Western Jess as an exploratory assay, there was a 137 kDa band at week 7 but none at baseline.

Abbreviations: ddPCR = droplet digital polymerase chain reaction; IF = immunofluorescence;

LLOQ = lower limit of quantitation; PDPF = percent dystrophin-positive fibers

**Reference**

1. Zaidman CM, Proud CM, McDonald CM, Lehman KJ, Goedeker NL, Mason S, Murphy AP, Guridi M, Wang S, Reid C, Darton E, Wandel C, Lewis S, Malhotra J, Griffin DA, Potter RA, Rodino-Klapac LR, Mendell JR (2023) Delandistrogene moxeparvovec gene therapy in ambulatory patients (aged ≥4 to <8 years) with Duchenne muscular dystrophy: 1-year interim results from Study SRP-9001-103 (ENDEAVOR). Ann Neurol 94:955-968. https://doi.org/10.1002/ana.26755
